# Soluble programmed death-ligand 1 rather than PD-L1 on tumor cells effectively predicts metastasis and prognosis in soft tissue sarcomas

**DOI:** 10.1038/s41598-020-65895-0

**Published:** 2020-06-03

**Authors:** Kunihiro Asanuma, Tomoki Nakamura, Akinobu Hayashi, Takayuki Okamoto, Takahiro Iino, Yumiko Asanuma, Tomohito Hagi, Kouji Kita, Kouichi Nakamura, Akihiro Sudo

**Affiliations:** 1 Department of Orthopedic Surgery, Mie University School of Medicine, Tsu City, Mie Japan; 2 Department of Pathology, Mie University School of Medicine, Tsu City, Mie Japan; 3 Department of Pharmacology, Faculty of Medicine, Shimane University, Izumo, Shimane Japan

**Keywords:** Sarcoma, Tumour biomarkers, Tumour immunology

## Abstract

The soluble form of PD-L1 (sPD-L1) is related to a poor prognosis in various cancers. Comparisons of sPD-L1 and PD-L1 expressed on tumor cells in soft tissue tumor patients have not been reported. The purpose of this study was to analyze serum sPD-L1 and PD-L1 levels in soft tissue tumor patients. A total of 135 patients with primary soft tissue tumors were enrolled in this study. The sPD-L1 level was quantitatively measured by enzyme immunoassay, and PD-L1 expression on high grade sarcoma cells was analyzed immunohistologically. There were no significant differences in sPD-L1 levels between benign (48) and soft tissue sarcoma (STS) patients (87). In STS, the high sPD-L1 (>44.26 pg/mL) group had significantly lower metastasis-free survival (MS) and lower overall survival (OS) than the low sPD-L1 group (≤44.26 pg/mL) at 5 years using the log-rank test. On multivariate Cox proportional hazard analysis, the high sPD-L1 group had significant differences in MS and OS compared to the low sPD-L1 group. Between positive and negative immunostaining groups, recurrence-free survival (RS), MS, and OS were not significantly different. No correlation was found between immunostaining and sPD-L1 with the Kappa coefficient. The sPD-L1 concentration could predict future metastasis and prognosis in STS patients. High sPD-L1 in STS patients may be a target for treatment with checkpoint inhibitors.

## Introduction

Soft tissue sarcomas (STSs), which are derived from heterogeneous malignant neoplasms arising in the mesenchymal connective tissues, comprise <1% of adult malignancies. Although the treatment approach, including surgery, radiotherapy, and combination chemotherapy has improved, more than 40% of cases have lethal postoperative metastatic recurrence^1^. Recently, attention has been focused on using immunological control points in the cell for immunotherapy in cancer. The immune response is usually in a balance between stimulatory and inhibitory signals. Programmed death-ligand 1 (PD-L1: B7-H1 or CD274), a 40-kDa transmembrane glycoprotein, is known as a primary ligand of PD-1. The interaction of PD-L1 and programmed death 1 (PD-1) can induce T-cell tolerance^2^, T-cell apoptosis^3^, and T-cell exhaustion^4^, leading to evasion of the host immune response and tumor aggravation. Some studies reported that high PD-L1 expression in tumor tissues was related to a poor prognosis in various malignant tumors, including non-small cell lung cancer^5^, ovarian cancer^6^, renal cell carcinoma^7^, melanoma^8^, breast cancer^9^, and STS^10^. Thus, it is recognized that PD-L1 expression affects tumor behavior and prognosis.

In addition, the soluble form of PD-L1 (sPD-L1) in blood has also attracted much attention. The associations of sPD-L1 with the clinical characteristics of various malignant tumors were studied, along with histological PD-L1 expression in tumor tissues. High sPD-L1 is related to a poor prognosis in various cancers, such as renal cell carcinoma^11^, hepatocellular carcinoma^12,13^, esophageal cancer^14^, lung cancer^15^, gastric cancer^16–18^, rectal cancer^19^, and lymphoma^20,21^. However, no study of sPD-L1 in soft tissue tumor patients and its relationship to prognosis has been reported.

The clinical data showing elevated sPD-L1 and a poor prognosis suggested that aggressive tumors may release and increase sPD-L1 or sPD-L1, making tumor cells aggressive. Given this, we hypothesized that there might be a relationship between the soluble sPD-L1 level and the prognosis of STS patients. The purpose of the present retrospective study was to evaluate correlations between serum sPD-L1 levels and clinicopathological parameters and to elucidate whether sPD-L1 levels and PD-L1 expressed on tumor cells can be used to distinguish the malignant phenotype in soft tissue tumor patients and to predict recurrence, metastasis, or prognosis in STS patients.

## Results

### Characteristics of the study population

The clinical and pathological characteristics of the study population are summarized in Table 1. Age and sPD-L1 levels were significantly different between healthy volunteers, the patients with benign tumors and the patients with STS. Although age distribution was different, sPD-L1 levels of STS were significantly high and those of healthy volunteers were low. Box plot of sPD-L1 was shown in Supplementary Fig. S1. The histopathological diagnoses of the 48 benign tumors were 17 lipomas, 15 schwannomas, 5 fibromatoses, 3 myxomas, 3 tenosynovial giant cell tumors, 2 leiomyomas, and 3 others, while those of the 87 STSs were 39 liposarcomas (23 well-differentiated liposarcomas (WLSs), 12 dedifferentiated liposarcomas (DLSs), and 4 myxoid liposarcomas (MLSs)), 14 myxofibrosarcomas (MFSs), 11 undifferentiated pleomorphic sarcomas (UPSs), 9 leiomyosarcomas (LMSs), 5 synovial sarcomas (SSs), 4 malignant peripheral nerve sheath tumors (MPNSTs), and 5 others. All patients with benign tumors underwent tumor resection, and 86 patients with STSs received treatment (wide resection 57 patients, marginal resection 24 patients, intralesional resection 3 patients, ion beam radiotherapy 2 patients) (Table 2). No treatment was performed for 1 patient with an MPNST; this patient was excluded from the prognostic analysis. Although female, patients over 60 years old and those with a history of other malignant tumors had higher sPD-L1 levels, there was no significant difference in sPD-L1 levels for characteristics in benign and STS patients (Table 1).

**Table 1:** Characteristics of patients with soft tissue tumors.

Characteristics		Healthy volunteers (10)	Benign (48)	STS (87)	p-value
Sex	Male	2	25	47	*0.126
	Female	8	23	40	
Age	Average (SD)	51.4 (12.5)	54.2 (13.7)	63.4 (15.1)	^#^P < 0.001
sPD-L1	Average (SD)	34.2 (10.3)	46.6 (24.7)	61.7 (58.2)	^#^0.017
**Characteristics in benign and STS patients**		**N (135)**	**sPD-L1 average(SD)**	**p-value**	
Sex	Male	72	55.0 (31.7)	**0.095	
	Female	63	57.7 (64.4)		
Age	≤60 y	61	48.4 (28.3)	**0.228	
	>60 y	74	62.8 (61.1)		
History of other malignant tumors	−	111	54.3 (36.1)	**0.324	
	+	24	65.3 (88.4)		

Sex, age, malignancy, and sPD-L1 values were evaluated by *Fisher’s exact test and the #Kruskal-Wallis test (upper table). sPD-L1 values were compared for each parameter by the **Mann-Whitney test (lower table).

**Table 2:** Characteristics of patients with STS.

Characteristics in STS patients		N (86)	sPD-L1 average (SD)	p-value
Sex	Male	47	55.2 (35.3)	*0.698
	Female	39	69.4 (77.3)	
Age	≤60 y	29	50.9 (32.7)	*0.342
	>60 y	57	67.2 (67.3)	
Tumor size	≤10 cm	42	58.6 (40.8)	*0.949
	>10 cm	44	64.6 (71.4)	
Location	Extremity	61	61.0 (60.9)	*0.977
	Trunk	25	63.3 (52.1)	
Tumor depth	Superficial	13	75.5 (62.2)	*0.539
	Deep	73	59.2 (57.6)	
Histological grade	Low grade	27	52.6 (40.9)	*0.098
	High grade	59	65.8 (64.5)	
Stage	I	27	52.6 (40.9)	^#^0.240
	II	15	59.8 (43.6)	
	III	44	67.8 (70.5)	
Treatment	Wide resection	57	63.7 (63.0)	^#^0.527
	Marginal resection	24	55.9 (43.3)	
	Intralesional resection	3	87.9 (92.8)	
	Ion beam radiotherapy:	2	32.0 (17.4)	
Chemotherapy	−	60	64.5 (65.7)	0.951
	+	26	54.9 (35.6)	
Radiotherapy	−	65	59.7 (60.7)	0.185
	+	21	67.7 (50.6)	
History of other malignant tumors	−	68	59.2 (40.6)	0.316
	+	18	70.8 (101.8)	

sPD-L1 values were compared for each parameter in STS patients. *Mann-Whitney test, ^#^Kruskal-Wallis test.

### Characteristics of the STS population

The clinical and pathological characteristics of the STS patients are shown in Table 2. The average sPD-L1 levels in STS patients were higher in females, those over 60 years old, with superficial tumors, with trunk tumors, or with histopathological high-grade tumors, but the differences were not significant. By histopathological subgroups, average (standard deviation) sPD-L1 levels were: MPNST 85.5 (74.2) pg/mL; MFS 86.2 (116.6) pg/mL; UPS 55.8 (30.1) pg/mL; SS 51.6 (14.9) pg/mL; WLS 54.4 (43.8) pg/mL; DLS 55.7 (30.2) pg/mL; MLS 44.8 (22.0) pg/mL; LMS 50.8 (19.0) pg/mL; and others 72.0 (64.8) pg/mL (Supplementary Fig. S2). According to the AJCC classification of STSs, 27 patients were classified as stage I, 15 were classified as stage II, and 44 were classified as stage III. The average sPD-L1concentrations tended to be higher with higher stages than with lower stages, but the difference was not significant.

### Recurrence, metastasis, and dead of disease in the STS group

The median follow-up in malignant patients was 42.9 months (range 1.1–417 months). During the period of this study, 18 patients developed recurrence (recurrence group), 28 patients developed metastasis (metastasis group), and 19 patients died of disease (DOD group). The recurrence group showed higher sPD-L1 concentrations than the no recurrence group, but the difference was not significant. The metastasis group and the DOD group had significantly higher sPD-L1 concentrations than the no metastasis group and the no DOD group, respectively (Table 3). Additionally, 10 cases were followed-up by measuring sPD-L1 concentrations at the operation for recurrence or metastasis. In 2 cases, sPD-L1 concentrations were decreased, and in 2 other cases, sPD-L1 concentrations were at almost the same levels. In the 6 other cases, sPD-L1 concentrations were increased by recurrence or metastasis (Supplementary Fig. S3).

**Table 3:** sPD-L1 levels in cases of recurrence, metastasis, or dead of disease.

Characteristic		n (86)	sPD-L1 average (SD)	p-value
Recurrence	−	68	61.7 (61.3)	0.803
	+	18	61.4 (46.4)	
Metastasis	−	58	50.2 (32.1)	0.003
	+	28	85.3 (87.3)	
Dead of disease	−	67	53.2 (35.5)	0.016
	+	19	91.5 (100.8)	

In the period of this study, 19 patients had recurrence, 28 had metastases, and 19 were DOD. The sPD-L1 levels of patients with metastasis and DOD patients were significantly higher than those of patients without metastasis and patients who were not DOD, respectively, by the Mann-Whitney test.

**Figure 1:**
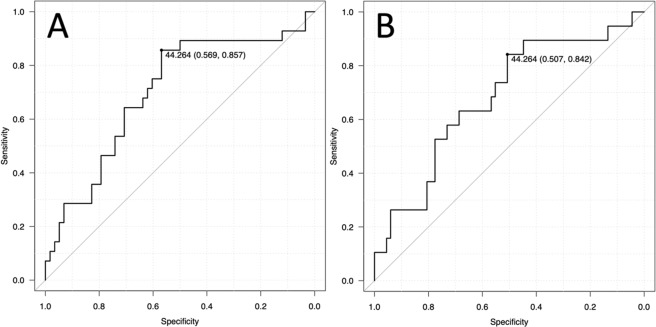
Receiver operating characteristic curve analysis. Diagnostic accuracy is evaluated by the area under the curve for identifying metastasis (**A**, AUC: 0.700, 95% CI: 0.579–0.822) and DOD (**B**, right, AUC: 0.682, 95% CI: 0.543–0.820). A cut-off of 44.26 pg/mL results in sensitivity of 85.7% and specificity of 56.9% for identifying metastasis and sensitivity of 84.2% and specificity of 50.7% for identifying DOD.

To confirm the diagnostic accuracy of sPD-L1 for identifying metastasis and DOD, ROC analysis was performed by evaluating the area under the curve (AUC). The AUCs for identifying metastasis and DOD were 0.700 (95%CI 0.579–0.822) and 0.682 (95%CI 0.543–0.820), respectively (Fig. 1A,B). Using an sPD-L1 threshold of 44.26 pg/mL based on Youden’s index, the sensitivity and specificity for identifying metastasis were 85.7% and 56.9%, respectively, and for DOD they were 84.2% and 50.7%, respectively. Based on the ROC analysis, a cut-off value of 44.26 pg/mL was used to divide the groups into low (≤44.26 pg/mL) and high (>44.26 pg/mL) sPD-L1 groups.

### Logistic regression

To examine the associations of multiple factors for identifying recurrence, metastasis, or DOD, multiple logistic regression analyses were performed. No factors were significant for diagnosing recurrence. An sPD-L1 concentration greater than 44.26 ng/mL was associated with a significantly increased risk of metastasis and DOD (metastasis: OR 8.92, 95%CI 2.63–30.0, P < 0.001; DOD: OR 5.84, 95%CI 1.43–23.9, P = 0.014). Sex and tumor size, depth, and location were not related to the risk of metastasis or DOD (Table 4), whereas only age was related to DOD.

**Table 4:** Multiple logistic regression analysis.

Characteristic	Recurrence			Metastasis			Dead of disease		
	OR	95%CI	p-value	OR	95%CI	p-value	OR	95%CI	p-value
Male	1.44	0.46–4.50	0.528	0.53	0.19–1.49	0.231	0.87	0.26–2.86	0.828
Age	1.02	0.98–1.07	0.289	1.01	0.97–1.04	0.688	1.07	1.01–1.13	**0.024**
Size	0.98	0.90–1.06	0.645	0.95	0.89–1.03	0.246	0.99	0.91–1.08	0.906
Superficial	2.28 × 10^-8^	/	0.992	0.70	0.16–3.07	0.645	0.23	0.02–2.32	0.215
Trunk	2.80	0.87–8.91	0.082	0.92	0.29–2.91	0.896	2.29	0.65–7.97	0.193
sPD-L1 > 44.26	1.72	0.54–5.49	0.355	8.92	2.63–30.0	**0.001**>	5.84	1.43–23.9	**0.014**

Multiple logistic regression analysis to identify recurrence, metastasis, or dead of disease is shown. The ORs of sPD-L1 values were significant only in metastasis and DOD.

### Prognostic analysis

**Figure 2:**
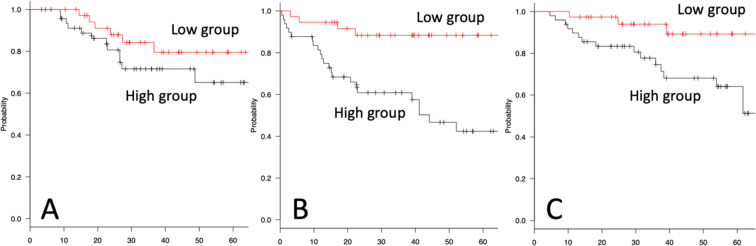
Kaplan-Meier analysis for STS. RS (**A**), MS (**B**), and OS (**C**) compared in the low- and high sPD-L1 groups are shown by Kaplan-Meier analysis. Five-year RS shows no significant difference (5 years: low sPD-L1 79.6%, high sPD-L1 65.1%, P = 0.205). The high-sPD-L1 group has significantly lower 5-year MS (low sPD-L1 88.4%, high sPD-L1 42.4%, p > 0.001). For 5-year OS, the high-sPD-L1 group has a significantly worse prognosis (low sPD-L1 = 89.2%, high sPD-L1 = 64.1%, P = 0.011). The X-axis indicates months.

Local recurrence-free survival (RS), metastasis-free survival (MS), and overall survival (OS) were compared between the low- and high sPD-L1 groups by Kaplan-Meier analysis and log-rank tests. RS showed no significant difference (5 years: low sPD-L1 79.6%, high sPD-L1 65.1%, P = 0.205) (Fig. 2A). In only high-grade tumors, RS showed no significant difference (5 years: low sPD-L1 = 64.5%, high sPD-L1 = 58.9%, P = 0.653, Supplementary Fig. S4A). The high-sPD-L1 group had significantly lower MS (5 years: low sPD-L1 88.4%, high sPD-L1 42.4%, P < 0.001) (Fig. 2B). In only high-grade tumors, MS showed a significant difference (5 years: low sPD-L1 = 79.9%, high sPD-L1 = 29.5%, P = 0.003, Supplementary Fig. S4B). For OS, the high-sPD-L1 group had a significantly worse prognosis (5 years: low sPD-L1 = 89.2%, high sPD-L1 = 64.1%, P = 0.011) (Fig. 2C). In only high-grade tumors, OS showed a significant difference (5 years: low sPD-L1 = 81.4%, high sPD-L1 = 5%, P = 0.040, Supplementary Fig. S4C), as did MS.

Furthermore, to adjust for the imbalance in prognostic factors among patients, multivariate Cox proportional hazard analysis was used. For RS, only trunk location showed a significant difference on multivariate analysis. For MS, only the high-sPD-L1 group showed a significant difference (HR 5.66, 95%CI 1.95–16.3, P = 0.001). For OS, the high-sPD-L1 group, in addition to age, showed a significant difference on multivariate analyses (HR 5.04, 95%CI 1.42–17.8, P = 0.012) (Table 5).

**Table 5:** Multivariate COX proportional hazard analysis.

	RS			MS			OS		
	HR	95%CI	p-value	HR	95%CI	p-value	HR	95%CI	p-value
Male	1.24	0.47–3.26	0.650	0.75	0.35–1.61	0.470	0.90	0.36–2.26	0.830
Age	1.03	0.98–1.07	0.150	1.01	0.98–1.04	0.300	1.07	1.02–1.12	**0.004**
Size	0.97	0.91–1.05	0.570	0.97	0.30–2.93	0.350	1.01	0.94–1.07	0.770
Super-ficial	3.71 10^-9^	/	0.990	0.94	0.30–2.93	0.920	0.31	0.04–2.44	0.260
Trunk	2.68	1.02–6.99	**0.043**	0.97	0.41–2.31	0.950	1.48	0.56–3.87	0.410
sPD-L1 > 44.2	1.98	0.72–5.42	0.170	5.66	1.95–16.3	**0.002**	5.04	1.42–17.8	**0.012**

For RS, only trunk lesions showed a significant HR. For MS, only high sPD-L1 showed a significant difference in the risk of metastasis. For OS, the HRs of sPD-L1 and age were significant.

### Immunohistology of PD-L1 for high-grade STS

PD-L1 immunohistological analysis was performed only for high-grade STSs. Of 59 high-grade STS cases, 6 cases were excluded due to tissue problems. Thus, a total of 53 patients were evaluated for histological positivity for cellular PD-L1 (more than 1% of membranous staining in tumor cells). Total positive staining for PD-L1 was observed in 16 patients (30.1%). The positive staining rate in each histological subtype was 41.7% in UPS, 27.3% in MFS, 20% in DLS, 66.7% in MLS, 12.5% in LMS, 25% in SS, and 33.3% in MPNST. The correlation between immunostaining and the sPD-L1 test was evaluated using the Kappa coefficient. All kappa values were below 0.351, and there was no correlation between PD-L1 immunostaining and the sPD-L1 test (Table 6). Furthermore, there was no significant difference in sPD-L1 levels between groups staining positive and negative for PD-L1 (positive: 52.8, negative 68.8, p = 0.437, Mann-Whitney test, Supplementary Fig. S5A). Between the positive and negative immunostaining groups, RS (5 years: positive 52.5%, negative 63.2%, P = 0.506), MS (5 years: positive 42.2%, negative 46.6%, P = 0.261), and OS (5 years: positive 51.6%, negative 55.0%, P = 0.511) were not significantly different on Kaplan-Meier analysis with the log-rank test (Supplementary Fig. S5B–D).

**Table 6:** Comparison between immunostaining for PD-L1 and sPD-L1 levels in high-grade STSs.

High-grade STS	n (53)	sPD-L1 group	PD-L1 immunostaining		Kappa coefficient
		High or low	+	−	
Total	53	High	9	22	−0.025
		Low	7	15	
UPS	12	High	4	3	0.351
		Low	1	4	
Myxofibrosarcoma	11	High	1	6	−0.294
		Low	2	2	
Liposarcoma (dedifferentiated)	10	High	0	5	−0.400
		Low	2	3	
Liposarcoma (myxoid)	3	High	1	0	0.400
		Low	1	1	
Leiomyosarcoma	8	High	1	4	0.157
		Low	0	3	
Synovial sarcoma	4	High	1	2	0.200
		Low	0	1	
MPNST	3	High	0	1	−0.500
		Low	1	1	
Others	2	High	1	1	0
		Low	0	0	

This table shows the comparison between positiv ity of immunostaining for PD-L1 and the level of high-sPD-L1 in high-grade STSs. Positivity of immunostaining for PD-L1 was defined as a positive stain rate of more than 1% of tumor cells. A high sPD-L1 was defined as a concentration greater than 44.26 pg/mL.

## Discussion

The expression of PD-L1 in tumor tissues of various malignant tumors has been reported. PD-L1 expression was observed not only in tumor cells, but also in active T cells, B cells, NK cells, DCs, monocytes, macrophages, activated vascular endothelial cells, and mesenchymal stem cells^22^. To date, the upregulation mechanism of PD-L1 mRNA or PD-L1 protein was found to be via cytokines such as IFN-γ, IL-4, IL-6, IL-10, VEGF, and hypoxia-inducible factor-1α. The signal pathway of IFN-γ/JAK2/IFN, PI3K, and MEK/ERK/STAT1 can upregulate them^23–25^.

Since PD-L1 is a transmembrane protein, the relationships between PD-L1 expression on various cells in tumor tissues and prognosis have been reported in several malignant tumors. A few studies of PD-L1 in STS were mainly histopathological analyses. Positivity of PD-L1 was seen in 2.5%^26^, 11.7%^27^, 12%^28^, 43%, and 64.8%^29^. In STS subtypes, positivity was seen as follows: UPS 14.9–82%; myxoid liposarcoma 0–73%; dedifferentiated liposarcoma 0–67%; synovial sarcoma 0–75%; leiomyosarcoma 0–70%; MPNST 0–50%; and myxofibrosarcoma 0–25%^27–30^. In the present study, positive staining for PD-L1 was observed in 16 patients (30.1%). PD-L1 positivity in STS subgroups varied widely. The present data for PD-L1 positivity were within the range previously reported. PD-L1 expression in tumor cells of STSs was not very intense. Some papers reported that PD-L1 positivity was a negative predictor of overall survival^10,27,29^, but other papers did not^26,31,32^. In the current study, there was no significant difference in prognosis between the PDL1-positive and PDL1-negative staining groups. Recently, in addition to cellular PD-L1, sPD-L1 has attracted attention, but there have been no reports about the prognosis in STS patients evaluated by serum sPD-L1 concentrations. The results of the present study successfully demonstrated the relationship between elevated sPD-L1 and a poor prognosis in STS. However, the sources of sPD-L1 were not elucidated.

Research into the source of sPD-L1 has progressed recently, and some important sources have been reported. First, one source is extracellular vesicles (EVs). Several studies reported PD-L1 on tumor-derived EVs including exosomes^33–37^. Cancer cells can secrete a majority of their PD-L1 on exosomes, even with only slight cellular PD-L1^36,37^. Basically, the amount of cellular PD-L1 and secretion of exosomal PD-L1 were different based on tumor cell types^33–37^. Interferon-γ could increase secretion of PD-L1, including exosomes^36^. Exosomal PD-L1 secretion may be controlled by the tumor environment, including stimulation by cytokines. The second source is spliced variants. Zhou et al. showed spliced variants that lack the transmembrane domain in the culture medium, and they were observed in the plasma of melanoma patients^38^. The third source of sPD-L1 is proteolytic cleavage of membrane PD-L1. Chen et al. reported that the release of sPD-L1 into culture supernatant was decreased by a metalloproteinase inhibitor^39^. This means that matrix metalloproteinase (MMP) may release PD-L1 from the cell membrane. Although these are potent sources of sPD-L1, the possibility of other sources of sPD-L1, such as cell stress, cell injury, or cell death, cannot be excluded. In the present study, there was no correlation between the sPD-L1 test and PD-L1 immunostaining. Thus, it was unlikely that the source of sPD-L1 was cellular PD-L1 in STS cells. However, PC3 cells (prostate cancer cell line) and WM164 (melanoma cell line) had only slight cellular PD-L1, but secreted abundant exosomal PD-L1, and exosomal PD-L1 was increased by interferon-γ^36,37^. Although cellular PD-L1 in STS cells was limited, STS cells could not be excluded as a source of elevated sPD-L1 by considering the additional effect of cytokines or the nature of STS cells.

Functional assessment of sPD-L1 is extremely important. Several studies supported the notion that exosomal PD-L1 inhibited IL-2 release and killing of tumor cells by T cells. Exosomal PD-L1 injection exacerbated transplanted tumor, and inhibiting the release of exosomal PD-L1 from tumor cells could decrease tumor growth^33,36,37^. Takeuchi et al. developed a unique ELISA to detect sPD-L1 that possessed binding capacity to PD-1 by using PD-1-Ig fusion protein for capturing sPD-L1^40^. This ELISA can differentiate sPD-L1 that binds membrane PD-1 from types that do not. Additionally, spliced variants of sPD-L1 show inhibitory functions on T-cell activation and proliferation^38^. Thus, the notion that circulating sPD-L1 has the potential to induce systemic immune suppression has been supported. In the present study, sPD-L1 had a strong relationship with metastasis and DOD in STS patients. Once the combination of high sPD-L1 and malignancy occurred, it led to worse MFS and OS in the high-sPD-L1 group than in the low-sPD-L1 group. An sPD-L1 > 44.26 pg/mL can predict future metastasis and a poor prognosis. High sPD-L1 was strongly related to metastasis and a poor prognosis. Thus, sPD-L1 may have potential to exacerbate tumor behavior in STS.

There have been some clinical trials of checkpoint inhibitors for sarcomas. In a small phase 2 study, six patients with synovial sarcoma were treated with ipilimumab. Time to progression ranged from 0.47 to 2.1 months (median 1.85 months), and overall survival was from 0.77 to 19.7 months (median 8.75 months)^41^. In another phase 2 study involving 80 patients with bone sarcomas or STSs treated with pembrolizumab, 7 (18%) of 40 patients with STSs and 2 (5%) of 40 patients with bone sarcomas showed objective responses^32^. In the most recent phase 2 study, 85 patients with bone sarcomas and STSs were treated with nivolumab with or without ipilimumab. The response rate was 5% in the nivolumab monotherapy group (43 patients) and 16% in the nivolumab and ipilimumab combination group (42 patients). Median overall survival was 10.7 months in the monotherapy group and 14.3 months in the combination group^42^. A clinical study of bone sarcoma and STS patients treated by checkpoint blockade therapy has just begun. These studies did not include evaluation of expressions of checkpoint molecules in the enrollment criteria. The authors pointed out the need to develop predictive biomarkers to establish which patients with sarcoma are most likely to benefit from checkpoint blockade, because, in the clinical data of treatment with checkpoint inhibitors, patients received benefits from this therapy regardless of PD-1 expression^32,42–46^. The present study showed that sPD-L1 concentrations could predict future metastasis and prognosis. Since sPD-L1 had a strong relationship with tumor aggravation, high sPD-L1 in STS patients may be a target for treatment by checkpoint inhibitors.

This retrospective study has some limitations. The number of patients was small, and subtypes could not be analyzed statistically because soft tissue tumors including sarcomas had over 20 histological subtypes, and the incidence rate of STS was low; thus, many studies must analyze STS as a whole entity, not by each histological classification. More blood samples within each histological subtype and longitudinal measurements may give us a more accurate assessment of the functional location of sPD-L1 in STS. We believe that the measurement of sPD-L1 may be useful for identifying metastases and poor outcomes in patients with STS.

## Materials and Methods

### Patients

A total of 135 patients with primary STSs who visited Mie University Hospital from 2009–2016 were enrolled in this study. Patients who had local recurrence or who were referred for additional resection after inadequate resection in a previous hospital or who had distant metastasis at the first visit were excluded from this study. Written, informed consent was obtained from each patient. For patients below the age 19 years, informed consent was obtained from their parents or legal guardian. This study was approved by the Ethics Committee of the Mie University Graduate School of Medicine. All procedures performed in studies involving human participants were in accordance with the ethical standards of the Ethics Committee of Mie University and with the Helsinki declaration of 1975. The histopathological diagnosis and histological grade were verified by independent pathologists. Clinical stage was classified according to the 7^th^ edition of the American Joint Committee on Cancer (AJCC) classification of STSs.

### sPD-L1 measurement

Blood samples were obtained from all patients before biopsy or treatment. To remove remaining cells, serum tubes were centrifuged at 1500 g for 10 min at 4 °C. The serum samples were aliquoted and stored at −80 °C.

Serum PD-L1 levels were measured quantitatively by enzyme immunoassay. On the measurement day, stored serum samples were thawed, and 100 µL of serum were used for further analysis. Levels of PD-L1 were measured using a commercially available sandwich enzyme-linked immunosorbent assay (Human PD-L1 ELISA Kit, ab214565, Abcam, Cambridge, MA) according to the manufacturer’s recommendations. The minimum detectable level of sPD-L1 was 2.91 pg/mL; values under the detectable level were assigned a value of 0 pg/mL.

### PD-L1 immunohistological analysis for high-grade STS

After being deparaffinized in xylene and rehydrated in alcohol, to retrieve the antigenicity of PD-L1, hydrated heating in 1 mM EDTA buffer (pH 8.0) was performed in a pressure cooker (Clipso 4 L; T-FAL, Rumily, France) for 10 min. After the sections were left at room temperature to cool in the soaking solution for 30 min, the sections were incubated with anti-PD-L1 (E1L3N) XP rabbit monoclonal antibody (CST, Danvers, MA) at a dilution of 1:200. Antibody was diluted in 1% BSA/TBS to suppress the nonspecific reaction. After washing with tris-buffered saline (TBS), endogenous peroxidase was inactivated by 0.3% hydrogen peroxide in methanol for 30 min. The sections were incubated with the reagent, peroxidase-labeled anti-rabbit immunoglobulin (DAKO, Glostrup, Denmark). The peroxidase was then intensified using fluorescyl-tyramide and anti-fluorescein conjugate HRP included in the CSA II Biotin-free Tyramide Signal Amplification System (DAKO). The reaction products were visualized in 0.15 mg/mL 3,3′-diaminobenzidine tetrahydrochloride (DAB) solution containing hydrogen peroxide. After washing in water, specimens were counterstained with hematoxylin. An individual pathologist evaluated PD-L1-positive cells. A tumor with membranous staining of more than 1% of tumor cells was considered positive for PD-L1 expression.

### Statistical analysis

Statistical analysis was performed to compare the serum sPD-L1 levels to various clinical parameters using the Mann-Whitney *U-*test or the Kruskal-Wallis test for quantitative data. To evaluate the threshold for detecting recurrence, metastasis, or death due to disease, receiver operating characteristic (ROC) curve analysis was performed. The ROC curves were created by plotting sensitivity on the y-axis and the false-positive rate (1-specificity) on the x-axis, and the area under the curve (AUC) was assessed. Local recurrence-free survival (RS) was defined as the time from the initial treatment to the date of clinically documented local recurrence. Metastasis-free survival (MS) was defined as the time from the initial treatment to the date of clinically documented distant metastasis. Overall survival (OS) was defined as the time from the initial treatment to the date of death attributed to the neoplasm. Kaplan-Meier survival plots and log-rank tests were used to assess the differences of RS, MS, and OS. The correlation between immunostaining and sPD-L1 test results was evaluated by the kappa coefficient test. To adjust for the imbalance in prognostic factors among patients, Cox proportional hazard analysis was used. P < 0.05 was considered significant. The EZR software program was used for statistical analyses^47^.

## Supplementary information

Supplementary information.
