# Supplementary material for: Soluble programmed death-ligand 1 rather than PD-L1 on tumor cells effectively predicts metastasis and prognosis in soft tissue sarcomas
Source: Sci Rep. 2020 Jun 3;10:9077. doi: 10.1038/s41598-020-65895-0 (PMC7270095; doi:10.1038/s41598-020-65895-0)
Supplement: Supplementary file 1 — Supplementary information. [file 41598_2020_65895_MOESM1_ESM.docx]

Soluble programmed death-ligand 1 rather than PD-L1 on tumor cells effectively predicts metastasis and prognosis in soft tissue sarcomas

^1^*Kunihiro Asanuma M.D., Ph.D., ^1^Tomoki Nakamura M.D., Ph.D., ^2^Akinobu Hayashi M.D., Ph.D., ^3^Takayuki Okamoto Ph.D., ^1^Takahiro Iino Ph.D., ^1^Yumiko Asanuma M.D., Ph.D. , ^1^Tomohito Hagi M.D., ^1^Kouji Kita M.D., ^1^Kouichi Nakamura M.D., ^1^Akihiro Sudo M.D., Ph.D.

1. Department of Orthopedic Surgery, Mie University School of Medicine, Tsu City, Mie, Japan
2. Department of Pathology, Mie University School of Medicine, Tsu City, Mie, Japan
3. Department of Pharmacology, Faculty of Medicine, Shimane University, Izumo, Shimane, Japan

**Corresponding author:** Kunihiro Asanuma

514-8507 Mie University, Orthopedic Surgery, 2-174 Edobashi, Tsu City, Mie, Japan

TEL: +81-59-231-5022, FAX: +81-59-231-5211

E-mail: [kasanum@gmail.com](mailto:kasanum@gmail.com)

**Key words:** PD-L1, soluble, soft tissue tumor, soft tissue sarcoma, metastasis, prognosis, soluble programmed death-ligand 1


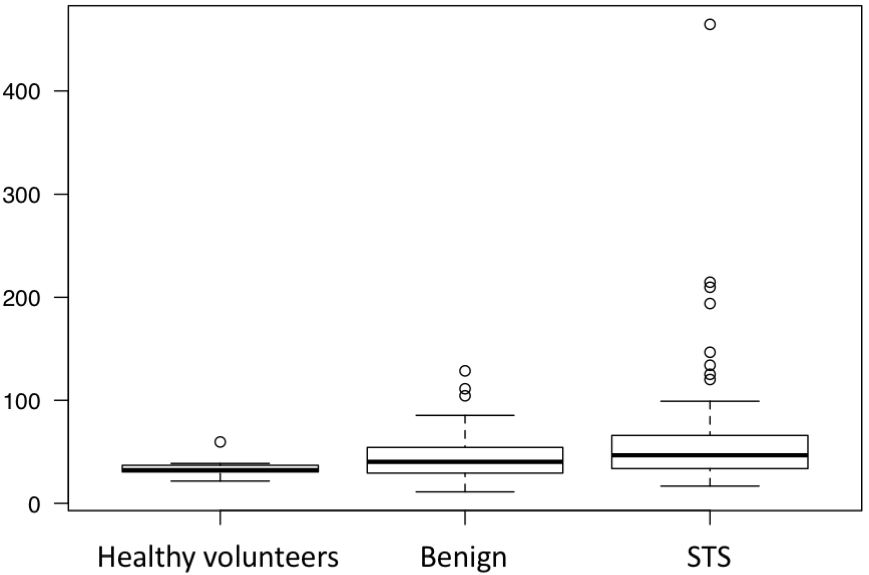


Supplementary Figure S1. Box plot of sPD-L1 levels

The difference of sPD-L1 levels between healthy volunteers, the patients with benign tumors and the patients with STS were shown.


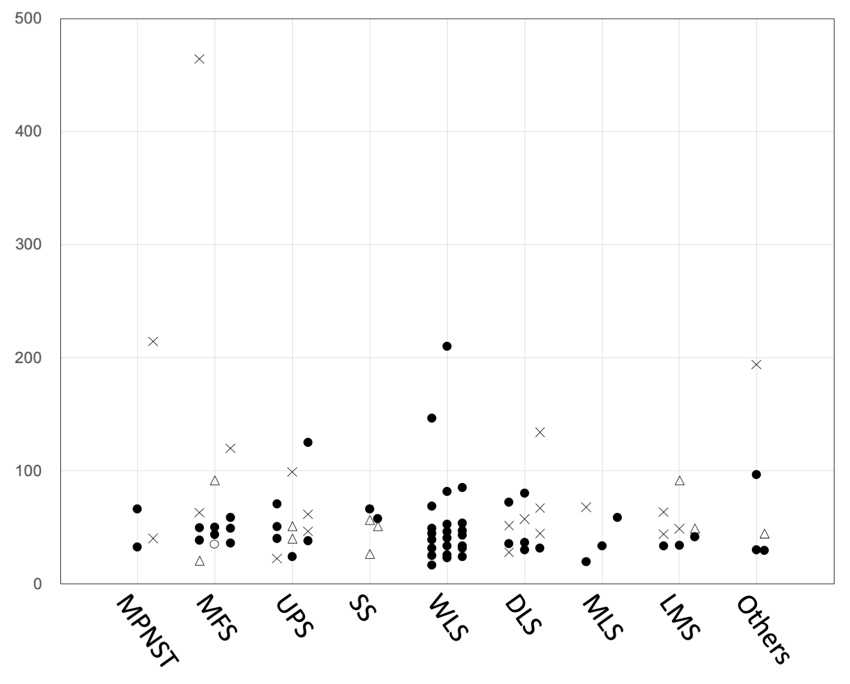


Supplementary Figure S2. Dot plot of sPD-L1 levels in histological subtypes of STS

Closed circles indicate live patients. Triangles indicate live patients after metastasis. X-marks indicate dead patients.


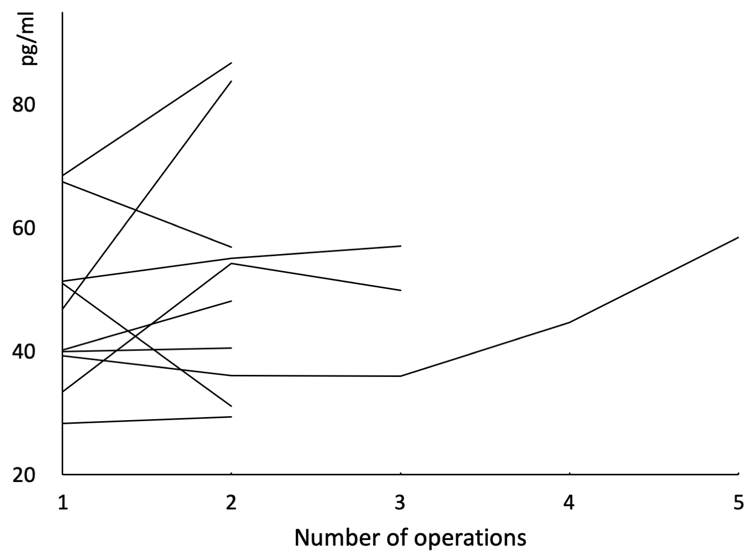


Supplementary Figure S3. sPD-L1 levels after recurrence of metastasis

sPD-L1 levels were measured at operation for recurrence of metastasis. 1 patient underwent 5 operations, and 2 patients underwent 3 operations after relapse.


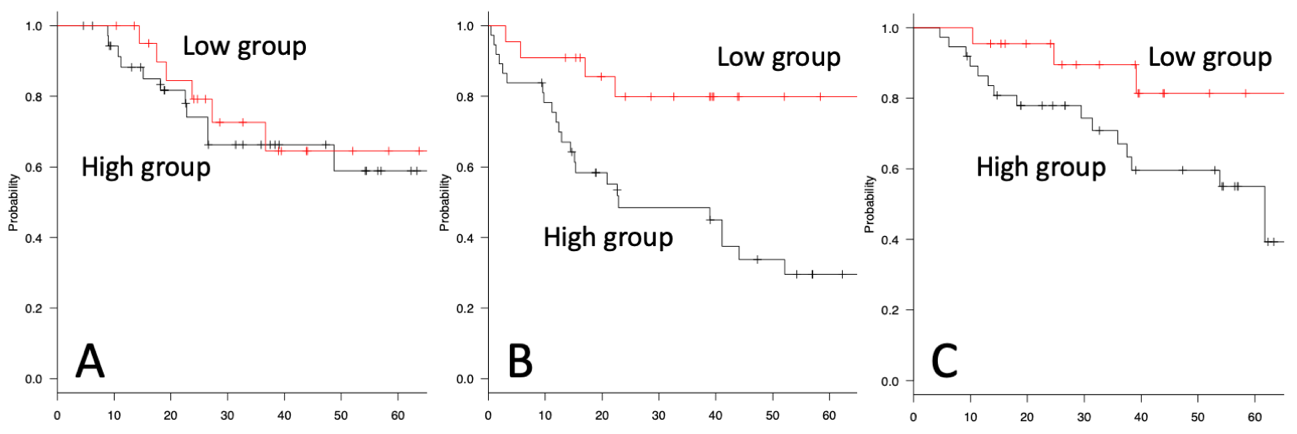


Supplementary Figure S4. Kaplan-Meier analysis for high-grade STS

In only high-grade tumors, RS shows no difference (5 years: low sPD-L1 = 64.5%, high sPD-L1 = 58.9%, P=0.653, A). MS shows a significant difference (5 years: low sPD-L1 = 79.9%, high sPD-L1 = 29.5%, P=0.003, B). OS shows a significant difference (5 years: low sPD-L1 = 81.4%, high sPD-L1 = 5%, P=0.040, C). The X-axis indicates months.


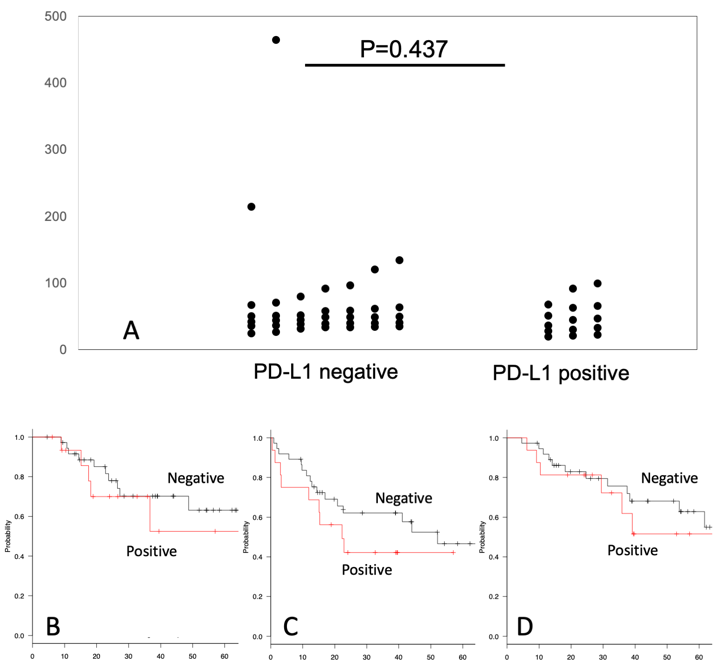


Supplementary Figure S5. Dot plot and Kaplan-Meier analysis between positive and negative staining for cellular PD-L1 in STS

Dot plot (A) shows no significant difference between positive and negative staining for cellular PD-L1. RS (B), MS (C), and OS (D) compared in the low- and high sPD-L1 groups are shown by Kaplan-Meier analysis between positive and negative staining groups. There are no significant differences.
